# Analysis of Huntington’s Disease Modifiers Using the Hyperbolic Mapping of the Protein Interaction Network

**DOI:** 10.3390/ijms23105853

**Published:** 2022-05-23

**Authors:** Aimilia-Christina Vagiona, Pablo Mier, Spyros Petrakis, Miguel A. Andrade-Navarro

**Affiliations:** 1 Institute of Organismic and Molecular Evolution, Faculty of Biology, Johannes Gutenberg University, Hans-Dieter-Hüsch-Weg 15, 55128 Mainz, Germany; avagiona@uni-mainz.de (A.-C.V.); munoz@uni-mainz.de (P.M.); 2 Institute of Applied Biosciences/Centre for Research and Technology Hellas, 57001 Thessaloniki, Greece; spetrak@certh.gr

**Keywords:** Huntington’s disease, paralogy, protein–protein interaction

## Abstract

Huntington’s disease (HD) is caused by the production of a mutant huntingtin (HTT) with an abnormally long poly-glutamine (polyQ) tract, forming aggregates and inclusions in neurons. Previous work by us and others has shown that an increase or decrease in polyQ-triggered aggregates can be passive simply due to the interaction of proteins with the aggregates. To search for proteins with active (functional) effects, which might be more effective in finding therapies and mechanisms of HD, we selected among the proteins that interact with HTT a total of 49 pairs of proteins that, while being paralogous to each other (and thus expected to have similar passive interaction with HTT), are located in different regions of the protein interaction network (suggesting participation in different pathways or complexes). Three of these 49 pairs contained members with opposite effects on HD, according to the literature. The negative members of the three pairs, MID1, IKBKG, and IKBKB, interact with PPP2CA and TUBB, which are known negative factors in HD, as well as with HSP90AA1 and RPS3. The positive members of the three pairs interact with HSPA9. Our results provide potential HD modifiers of functional relevance and reveal the dynamic aspect of paralog evolution within the interaction network.

## 1. Introduction

Huntington’s disease (HD) is one of nine autosomal dominant neurodegenerative disorders caused by the expansion of a CAG trinucleotide repeat. For HD, this expansion is located in the first exon of the huntingtin gene (htt) and results in an abnormally long poly-glutamine (polyQ) tract within the N-terminus of the huntingtin protein [1]. Expansion of CAG repeats results in the production of mutant proteins, which aggregate and form inclusions within neurons [2]. PolyQs with lengths above 40 amino acids cause mutant HTT proteins to misfold, form aggregates, become toxic, and cause disease [3].

The mechanism of polyQ-mediated toxicity is still under study; however, there is evidence supporting aberrant protein–protein interactions in the pathogenesis of HD [4,5,6]. Several lines of evidence support that expanded HTT is processed into N-terminal fragments that form inclusions in the cytoplasm and nucleus [7,8]. Many proteins, such as ubiquitin, heat shock proteins, and transcription factors, localize to polyQ inclusions [9,10,11].

Reports are accumulating on a variety of positive or negative effects that the expression or inhibition of multiple proteins have on HD’s progression or its effects [12,13,14,15,16,17,18]. While these positive or negative effects may be actively due to specific functions (e.g., the phosphorylation of particular residues in HTT [19,20]), previous work suggests that the interactions of proteins with polyQ-caused aggregates can passively trigger both the increase and decrease of aggregates [21,22].

We hypothesized that, given the large size of HTT and its large number of interactors [4], it should be possible to explore the relatively complex network of the interactions surrounding HTT. We previously exploited this possibility to demonstrate the existence of multiple partners of HTT that use similar modes of interaction [23]. Here, we reasoned that paralogous expansions in the set of proteins interacting with HTT with divergent effects could be used to pinpoint active functions with relevance in HD. It is well known that gene duplication and speciation events, followed by mutation, can lead to functional changes, meaning that proteins with high sequence similarity may not have the same function [24,25]. In particular, several lines of work report the opposite effect of pairs of paralogs, revealing a functional diversity [26]. Favaro et al. found that two very similar proteins, PSD-93, and its paralog PSD-95, although they share similar functional domains and have evolved through the duplication of a single ancestral gene, have opposite roles in glutamatergic synapse maturation [27].

We assumed that the identification of pairs of paralogs interacting with HTT with opposite effects on HD might reveal active functions relevant to HD, under the assumption that these paralogs might interact identically with HTT, but their different effect on HD would arise from different interactions with other functional components of the protein interaction network. To maximize the divergence in protein interactions, we would need to account for the entire human protein interaction network (hPIN) in an unbiased approach. This is facilitated by techniques that project networks in a geometric space where closeness means a higher connection probability.

Since proteins are very complex machines, and their interactions with other proteins form a very complicated molecular system, their study as a protein–protein interaction network has gained traction in recent years [28]. Several algorithms and models support the existence of a hidden geometry underlying the structure and topology of complex systems, such as the human protein–protein interaction network [29]. The Popularity-Similarity (PS) model assumes that clustering and the hierarchy of complex networks arise from trade-offs between node popularity and similarity [30]. Additionally, Alanis-Lobato et al. found that the embedding of the hPIN to hyperbolic space has biological interpretations in terms of the PS model. They realized that the radial positioning of the nodes encapsulates information about protein conservation and evolution, while their angular positioning captures the functional and spatial organization of proteins in the cell [29]. This mapping may also lead to a better understanding of complex human disorders [31].

Motivated by these results, we followed a step-by-step computational filtering strategy, starting from a large protein–protein interaction (PPI) dataset embedded in the hyperbolic disc to obtain a network that consists of HTT interactors. This mapping enabled us to select pairs of paralogs of HTT interactors located in different regions of the hPIN. Proteins in each of these pairs are expected to interact similarly with HTT, but their different positions in the hPIN suggest their different involvement in pathways or complexes. The evaluation of protein pairs with opposed effects on HD was interpreted to find common partners for positive or negative effectors, which we propose as potential candidates for powerful effects in HD models.

## 2. Results

### 2.1. Human Protein Interaction Network Embedding to Hyperbolic Disc (hPIN)

In the first step of our analysis, we created a protein–protein interaction network from the HIPPIE database with high-quality interactions formed with a confidence score of ≥0.71 [32,33]. The largest connected component (LCC) of the hPIN is comprised of 93,140 interactions between 13,076 proteins. The resulting network was embedded into the two-dimensional hyperbolic plane H^2^ using LaBNE+HM [34,35,36], and the hyperbolic coordinates were inferred for each protein of the network (Supplementary Table S1). We then proceeded to analyze the topological and geometrical properties of the hPIN.

### 2.2. Identification of Protein Clusters in the Angular Dimension

**Figure 1:**
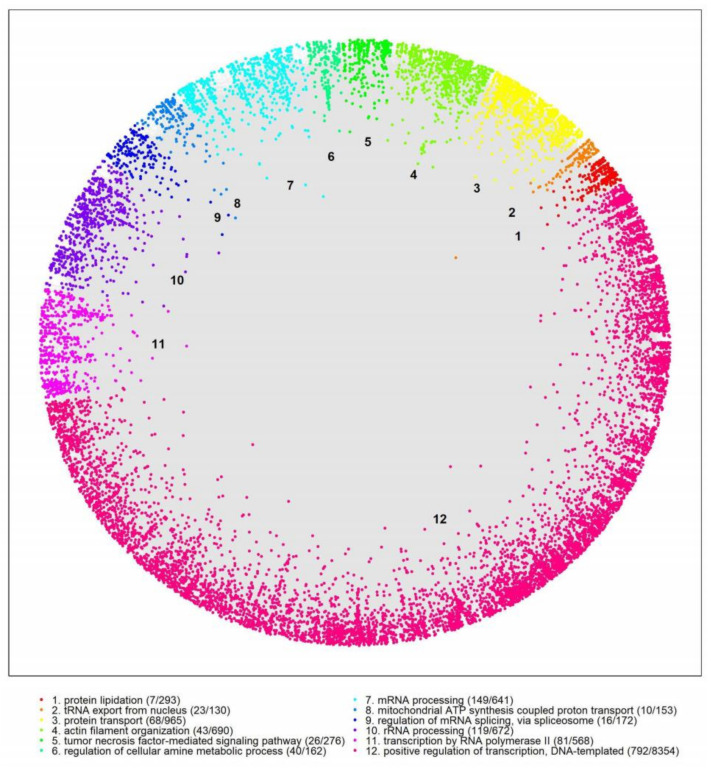
Human protein–protein interaction network embedded in the hyperbolic disc. Protein clusters in different colors were identified by big gaps separating groups of proteins in the angular dimension of the hyperbolic space. The overrepresented biological function in each cluster was determined via GO enrichment analysis (BP: Biological Process). Each cluster was assigned a numeric identifier (1–12). For each protein cluster, the number of proteins that are associated with the GO BP terms and the number of proteins in each cluster are shown.

The similarity component of the PSM (angular coordinates of the nodes in H^2^) abstracts the characteristics that make a node similar to others [29]; neighboring proteins play a role in similar biological processes [31]. To explore the biological meaning of the angular dimension, we identified big gaps between consecutive inferred angles and determined 12 protein clusters in the hPIN (Figure 1; Supplementary Figure S1; see Section 4 for details). From the biological point of view, the angles capture the functional organization of the cell, supported by the GO term annotations of the proteins in each cluster. As an example, the overrepresented biological process of cluster 1 is *protein lipidation*. Proteins agglomerate in similarity-based clusters since each of them is enriched in different aspects of the GO BP terms.

### 2.3. HTT-Interactors in the Hyperbolic Disc

**Figure 2:**
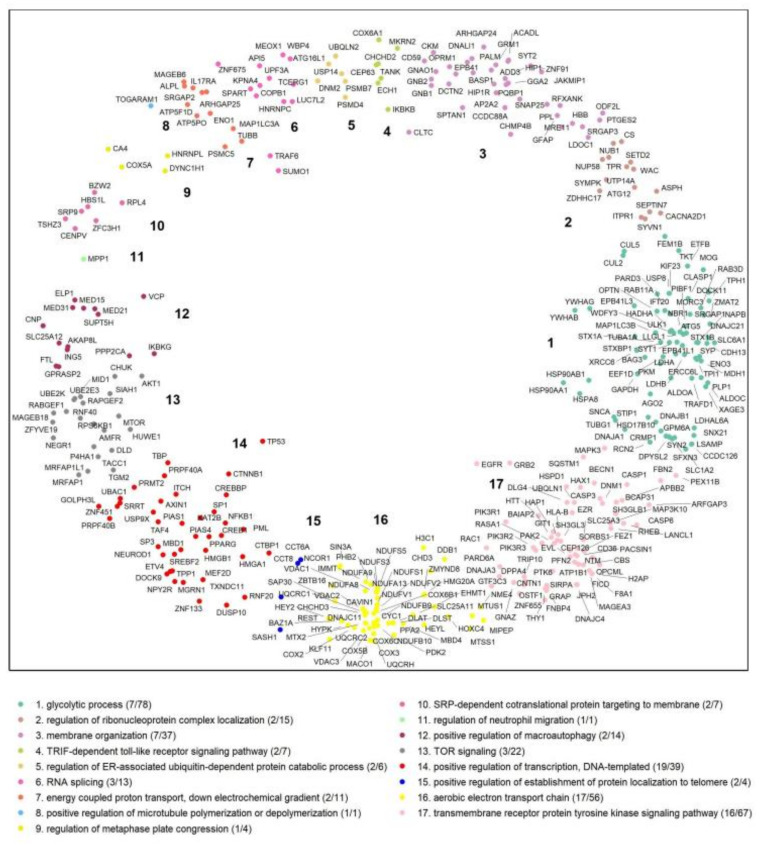
HTT interactors in the H^2^. The different clusters were identified by big gaps separating groups of proteins in the angular dimension of the hyperbolic space. Each cluster was assigned a numeric identifier (1–17). The number of proteins/genes that are associated with the GO BP terms and the number of proteins/genes in each cluster are shown.

Starting from a large human protein interaction network with 13,076 proteins, we performed an interaction network filtering procedure in order to limit the dataset to a relatively small network, focusing on the HTT protein and its interactors (HttPIN). We observed, in this network, 382 proteins that are directly linked to HTT (Figure 2; Supplementary Table S2).

To reveal the functional modularity of the HTT interactors, following the procedure used above to cluster the hPIN, we partitioned the angular dimension of the nodes of the HttPIN into several sectors according to large gaps between the consecutive inferred angles of the HTT interactors (Supplementary Figure S2; see Section 4 for details). As it is shown in Figure 2, proteins agglomerate into 17 sectors. The overrepresented biological function in each cluster was determined through GO enrichment analysis and points to the heterogeneity of the clusters, since no common GO BP terms were observed between the sectors.

### 2.4. Paralog Pairs of HTT Interactors

**Figure 3:**
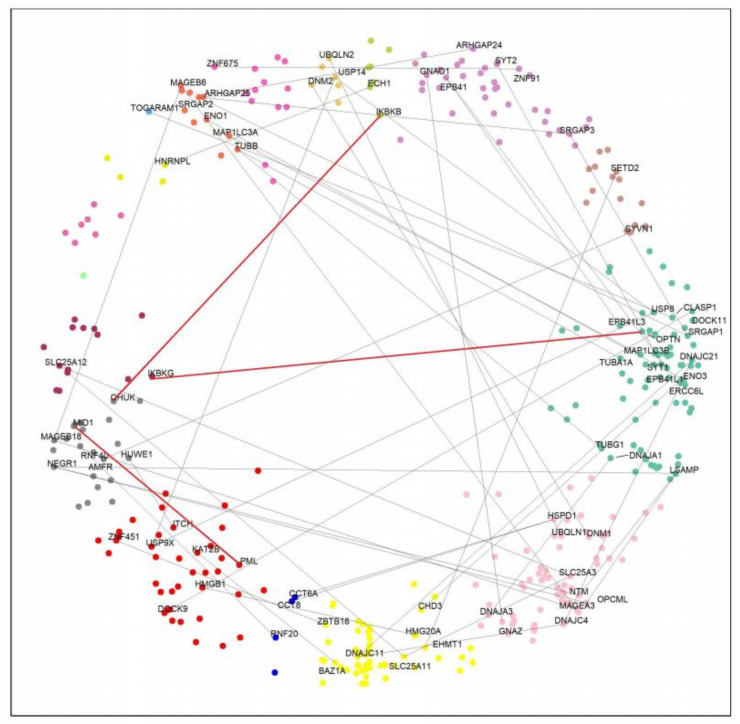
Paralog pairs of HTT interactors in different clusters. Nodes in different colors display protein agglomeration in angular similarity-based sectors. The nodes with the Gene Symbol represent the 49 paralog pairs located in different clusters. Paralog pairs are connected by an edge. Red edges indicate the three pairs of paralogs with opposite effects on HD (see text for details).

To further investigate our hypothesis, we looked for HTT interactors with paralogs having opposite effects on HD. Previous computational analysis in yeast highlighted the value of studying protein–protein interaction networks to examine the functional divergence among duplicated gene products [37,38]. From all 382 HTT interactors, we obtained 87 paralogous pairs (Supplementary Table S3). Considering that the geometrical properties of the hyperbolic disc capture biologically relevant features, such as function, we speculated that paralogous proteins in different clusters could have divergent effects on the disease. Therefore, we selected paralog pairs of proteins detected in different clusters (Supplementary Table S4). Figure 3 shows this network, which consists of 74 nodes and 49 paralog pairs. Overall, 87 paralog pairs interact with HTT (Supplementary Table S3); 49 of them are located in different clusters, while 38 are in the same cluster.

### 2.5. Effects of Paralog Pairs on HD

We then reviewed the literature to find paralog pairs with opposite effects on HD (Supplementary Table S4). More specifically, DNAJC21 and its paralogs: DNAJC11, DNAJC4, DNAJA1, and DNAJA3 are all members of the DnaJ heat shock protein family (Hsp40). Previous studies in animal models have shown that Hsp40 chaperones are protective of neurodegeneration [39]. The overexpression of Hsp40 proteins can suppress polyQ aggregation, and, hence, they are critical for cell survival [40,41]. In addition, CCT8 and CCT6A, which are paralog proteins of HSPD1, have a protective role on HD. In fact, the upregulation of CCT8 has been linked to a mechanism that protects from polyQ aggregation, while the knockdown of CCT6A led to stimulating the aggregation of expanded polyglutamine and mutant huntingtin in cellular models [42,43]. Moreover, an increase in the UBQLN1 expression protects against HTT-polyQ-induced cell death and toxicity. Likewise, UBQLN2 significantly decreases in both wild-type and polyglutamine-expanded full-length HTT levels in cellular and animal models [12]. Finally, the TUBB protein interacts more strongly with mutant HTT than with the wild-type. This event blocks intracellular transport, suggesting a pathogenetic mechanism in HD [16].

We next focused on pairs of paralogs located in different regions of the hPIN and with opposite effects on HD according to the literature (Table 1).

We detected three paralog pairs with opposite roles on HD. Notably, MID1/PML, IKBKB/IKKA(CHUK), and IKBKG/OPTN are paralogous pairs with experimental evidence suggesting their different effect on HD. MID1 leads to an aberrant overproduction of the mutant polyglutamine protein, inhibition of IKBKB has a protective effect on neurodegeneration, and IKBKG binds mutant HTT contributing to HD neurotoxicity [46,49,51]. The potential participation of IKBKB and IKBKG in the pathogenesis of HD was discussed in a computational analysis [52]. Differently, several lines of evidence support that PML, IKKA, and OPTN play a protective role against neuronal toxicity associated with HD [45,48,49,50].

### 2.6. Common Interactors between Positive and Negative Paralogs

**Figure 4:**
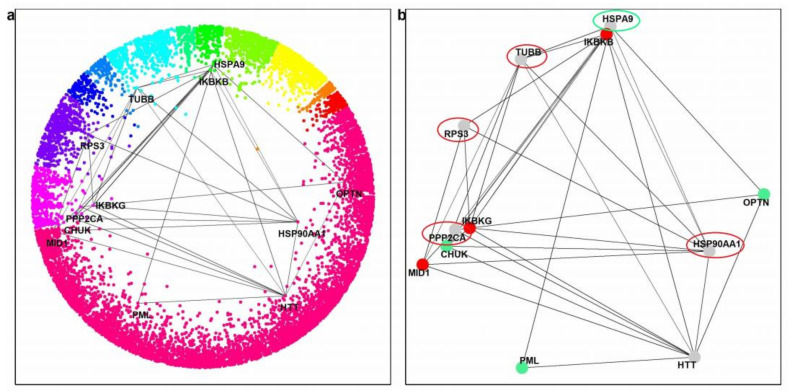
Positive and negative effectors on HD and closely connected components. (**a**) Position of the positive and negative paralogs and their common interacting partners in the hyperbolic disc. (**b**) Red nodes represent the negative paralogs, and the nodes surrounded by the red ellipses are their common interactors. Green nodes represent the positive paralogs, and the node surrounded by the green ellipse represents their common interactor.

Finally, we hypothesized that the existence of common interaction partners of the three negative paralogs could reveal functions that negatively influence HD and whose inhibition could have therapeutic effects. The three negative paralogs are IKBKB, IKBKG, and MID1. Using the HIPPIE database, we obtained their common interactors, besides HTT. Filtering interactions with a confidence score of ≥0.71 resulted in only PPP2CA. At a confidence score of ≥0.63, TUBB, HSP90AA1, and RPS3 were also found (see Figure 4).

We used the same approach with all three positive partners to find proteins that might be effective as a therapy for HD. The three positive paralogs are OPTN, PML, and CHUK. Using the HIPPIE database and a confidence score of ≥0.49, besides HTT, only one partner was found, chaperon HSPA9 (also known as mortalin), which is not a direct interactor of HTT (Figure 4).

Finally, we checked the connectivity of the common interacting partners of the paralogs with HTT. All five (including RPS3 and HSPA9, which do not bind directly to HTT) rank very high on the list of human proteins ordered by the number of interactions with HTT-interactors (among the top 4% of 10,914 ranked proteins; Table 2).

## 3. Discussion

The two-dimensional hyperbolic embedding of the human protein interaction network has been shown to be both relevant and useful. We previously showed (i) that the radial coordinates of nodes (proteins) correlate with protein age, with older proteins occupying more central positions, and (ii) that proteins with related biological functions and cellular localizations cluster together along the angular coordinates [29]. The reason for these distributions, in terms of the protein interaction network, is that older proteins have more interactions, and their corresponding nodes are shifted towards the center of the map, while proteins with similar functions tend to be part of the same complexes and pathways and, therefore, because they interact or have common interactors, they tend to be pulled together towards the same region of the map.

In this paper, the latent geometry of the hPIN proves useful, namely in the network-based analysis of huntingtin’s interactors. Considering pairs of paralogs that (a) both interact with huntingtin, (b) are located in different regions of the hPIN, and (c) have opposite effects on HD, we found three pairs that correspond to these criteria. Particularly, MID1 is an aberrant interaction partner of HTT. Its binding leads to the induction of an aberrant translation of the mutant HTT mRNA. MID1 assembles a protein complex with its interaction partners, PP2A and 40S ribosomal S6 kinase (S6K), and recruits this complex to the mutant HTT mRNA. This recruitment induces translation in a CAG repeat length-dependent manner, resulting in a toxic gain of function [46]. The translational induction by MID1 has also been found in models of other CAG repeat diseases [53]. Heinz and colleagues found that blocking the interaction between MID1 and the mutant HTT mRNA is a promising therapeutic approach [13]. On the other hand, the MID1 paralog protein, PML, can associate directly with polyQ proteins and preferentially with the pathogenic form, recognizing structures or regions that are commonly found in misfolded proteins [45]. Misfolded nuclear proteins that are selectively recognized by PML are marked with poly-SUMO2/SUMO3 chains. RNF4, which is a SUMO-dependent E3 ubiquitin ligase, binds to the poly-SUMO2/SUMO3 chains via tandem SUMO interacting motifs (SIM) and ubiquitylates, the protein, which leads to its proteasomal degradation [44]. This relay system likely provides a critical link between misfolded proteins and may play an important role in protecting against neurodegeneration.

Another paralog pair with opposite effects on HD is IKBKB/CHUK(IKKA). Most of the IKKA and IKBKB molecules in the cell are part of IKK complexes. The IKK complex also contains a regulatory subunit called IKKγ or NEMO [54]. Concurrently, DNA damage is an important factor in the development of neurotoxicity and a potential regulator of HD pathology [55]. It was shown that the induction of DNA damage has opposite effects on this paralog pair, increasing the activity of IKBKB while decreasing the activity of IKKA in the neurons [47]. The increased activity of IKBKB is also involved in several neurodegenerative disorders, including HD, Alzheimer’s disease (AD), and Parkinson’s disease (PD) [49,56,57]. The IKBKB activation by the DNA damage promotes HTT cleavage, and by increasing IKKA or reducing IKBKB, blocks this event. In the context of neuronal DNA damage, IKBKB activation is deleterious, and its inhibition may be protective in HD and potentially in other neurodegenerative disorders where DNA damage plays a role [47]. Moreover, the inactivation of IKBKB prevents the development of metabolic abnormalities induced by mutant HTT in the hypothalamus [58].

IKBKG, which is the regulatory module of the IKK complex, binds to mutant HTT through polyQ and polyP regions. This binding activates the IKK complex and promotes the activation and nuclear localization of the nuclear factor kappa (NF-κB) [49]. Activated NF-κB is involved in neuronal injury and in pathological conditions, such as HD [59,60]. The inhibition of NF-κB may have a protective effect on excitotoxicity, apoptosis, and neurodegeneration and, therefore, NF-κB inhibitors may deserve investigation for their potential role in HD [51]. Optineurin (OPTN) is one of a number of HTT-interacting proteins [4] that promotes neuronal survival by counteracting the glutamate-induced neurotoxicity in diseases, demonstrating its neuroprotective role [48,61]. Shen and colleagues also showed that OPTN decreased the misfolded protein aggregates, mainly through polyUbK63-linked autophagy, while OPTN mutations lead to diseases by altering the protein quality control and degradation machinery [50].

We next focus on common partners of the three negative proteins: TUBB, PPP2CA, HSP90AA1, and RPS3. It is interesting to note that TUBB is part of our paralog interacting set with a negative effect [16]. Furthermore, PPP2CA, HSP90AA1, and RPS3 are involved in the pathogenesis of another polyQ disease named SCA1. These proteins are members of a protein–protein interaction network, which is affected by the gradual aggregation of the relevant polyQ-expanded protein, ataxin-1, and the degeneration of Purkinje neurons in animal models [62]. HSP90AA1 interacts with the N-terminal of HTT and recruits the deubiquinating enzyme USP19 [13]. Additionally, this protein participates in a chaperome network, safeguarding proteostasis, and is repressed in the brain of patients with neurodegenerative diseases, including HD [63].

PPP2CA is an interesting candidate because it dephosphorylates S421 in HTT and, blocking its activity, was found to protect striatal neurons from NMDA-induced cell death [64]. This protein not only regulates translation of HTT mRNAs through the MID1-PP2A complex [46] but may also induce apoptotic cell death through the activation of the mTOR/PI3K/Akt pathway [65]. The selection of the ribosomal protein RPS3 in this network suggests a new avenue for exploration. Ribosomal proteins preferentially interact with the mutant HTT [66], suggesting the participation of the protein translation machinery in the pathogenesis of polyQ diseases [67]. The shuttling of RPS3 from the cytoplasm to the nucleus can be induced by toxic DNA damage [68] and to mitochondria by increased ROS levels [69]. Using the same approach with the three positive proteins, we found only one common partner: chaperone HSPA9 (also known as mortalin), which is not a direct interactor of HTT. However, its positive effect may be mediated by other members of the heat shock protein 70 family, including HSPA8, which was previously shown to preferentially interact with HTT [66]. The interactions of HSPA9 with OPTN, PML, and CHUK are reported by non-specific works [70,71,72] but could also suggest a positive effect. One high-throughput non-specific interaction study links it also to the negative HD modifiers, IKBKB, IKBKG, and TUBB [71]. We take recent work linking HSPA9 to roles in the control of peroxisomal function [73] and neuronal stress detection [74] and its downregulation in animal models of Alzheimer’s disease and patient’s brains [75] as a suggestion that this could be a relevant protein for the control of HD.

## 4. Materials and Methods

### 4.1. Construction of the hPIN

The hPIN is a subset of the Human Integrated Protein–Protein Interaction rEference (HIPPIE) [32,33]. HIPPIE retrieves interactions between human proteins from major expert-curated databases and calculates a score for each one, reflecting its combined experimental evidence. This score is a combination of the number of studies that detect an interaction, the quality of experimental techniques used to measure an interaction, and the number of non-human organisms in which an interaction was reproduced. The raw version of this network is available in the Download section of the HIPPIE database [32,33]. In this study, only interactions with a confidence score of ≥0.71 that belong to the largest connected component (LCC) in release 2.2 were considered (N = 13,076 nodes and L = 93,140 edges). The 0.71-network was preferred because it has a high percentage of edges supported by more than one experiment (70%).

### 4.2. Mapping the hPIN to Hyperbolic Space

In order to embed the hPIN into the two-dimensional hyperbolic plane, we used the R package “NetHypGeom,” which implements the LaBNE + HM algorithm [35]. This algorithm combines manifold learning [34,35] and maximum likelihood estimation [36] to uncover the hidden geometry of complex networks. The PS model has a geometrical interpretation in hyperbolic space (H^2^), where nodes that join the system connect with the existing ones that are hyperbolically closest to them [30,36,76]. The N nodes of the network lie within a hyperbolic disc with a radius of R~N, where the radial coordinate of a node, r_i_, represents the popularity dimension with nodes that joined the system first being close to the disc’s center. The angular coordinate, θ_i_, represents the similarity dimension. The network was embedded in the two-dimensional hyperbolic plane using the LaBNE + HM algorithm to infer the hyperbolic coordinates of each protein, with parameters *γ* = 2.74, T = 0.8, and *w* = 2π.

### 4.3. Clustering in the Similarity Dimension

To cluster proteins in the similarity dimension, we sorted the nodes increasingly by their angular coordinates and computed the difference between θ_i_ and θ_i+1_ to identify large gaps between groups of proteins. The gap size, *g*, that was chosen to separate protein clusters produces sectors with a minimum of ten components (*g* = 0.011344, see Supplementary Figure S1). Then, we applied an ad hoc rule, where clusters with less than 100 proteins were merged clockwise with the consecutive one to avoid redundancy. We then carried out Gene Ontology (GO) enrichment analysis [77] for the proteins in each sector of the hPIN, using the nodes of the hPIN as our background set. Only GO Biological Process (BP) terms enriched at the 0.05 significance level (*p*-value) were kept.

### 4.4. HTT Interactors in the Hyperbolic Space

We then obtained the list of human HTT interactors from the HIPPIEv2.2 database [32,33] and identified their position in the hyperbolic disc. We created groups of proteins in the HttPIN based on the angular similarity dimension of the HTT interactors. To determine the start and the end of each group, proteins were sorted increasingly by their inferred angular coordinate, θ, and the difference between θ_i_ and θ_i+1_ was computed. The gap size = 0.059198 was chosen (Supplementary Figure S2). The enriched GO BP terms for each group were determined and the ones enriched at the 0.05 significance level (*p*-values) were extracted.

### 4.5. Paralog Pairs and Common Interacting Partners

From the HTT interacting proteins dataset, we detected pairs of paralogs. This information was derived from the Ensembl BioMart database [78], using the human genome assembly, GRCh38.p13. We focused on pairs of paralogous proteins located in different clusters in the H^2^ to explore functional interpretations based on the angular similarity dimension. We then conducted a literature review to identify pairs with opposite effects on HD. For the latter analysis, we used the HIPPIEv2.2 database [32,33] to obtain common interacting partners between pairs of paralogs with negative and positive effects on HD, applying different confidence scores.

## 5. Conclusions

We approached the heterogeneity of the measurements of the effects of various proteins in HD models by providing a common framework for evaluation. Our hypothesis is that strong HD modifiers should produce collective effects through multiple pathways and complexes. These strong functional effectors may be obscured by the supposedly abundant but weaker effects of proteins that influence HD aggregates by their passive interaction with HTT. To avoid this problem, we focused on pairs of HTT interactor paralogs occupying divergent positions in the protein interaction network mapped to H^2^ and found pairs with opposite effects on HD. We then explored the components closely connected to the positive or negative effectors. Our findings confirm proteins with relevant effects in HD and suggest RPS3 and HSPA9 as non-direct interactors of HTT that could have a negative and positive effect in HD, respectively. With our approach, we have shown how the interaction network connects the effects of HD modifiers to the literature, and the finer details of each experiment can ultimately be examined to make sense of these results and select or discard ideas for experimental work.

## Figures and Tables

**Table 1:** List of paralog pairs with opposite effects on HD.

Genes	Proteins	Effects on HD	References
MID1/PML	E3 ubiquitin-protein ligase Midline-1/Protein PML	Mutant HTT recruits MID1 protein complex resulting in overproduction of polyQ HTT; PML plays a protective role against neuronal toxicity associated with polyQ proteins.	[13,44,45,46]
IKBKB/IKKA(CHUK)	Inhibitor of nuclear factor kappa B kinase subunit beta/Inhibitor of nuclear factor kappa-B kinase subunit alpha	Inhibition of IKBKB may promote neuronal survival in HD; IKKA has a protective role in preventing HTT proteolysis.	[47]
IKBKG/OPTN	NF-kappa-B essential modulator/Optineurin	Inhibition of IKBKG activity reduces HTT-polyQ toxicity; OPTN has a protective effect on polyQ neurotoxicity associated with mutant HTT.	[48,49,50]

**Table 2:** Common interacting partners between negative and positive paralogs.

Paralogs	Effect on HD	Common Interactors between Paralogs	Confidence Score	Interaction of Common Interactors with HTT	Ranking by # of Interactions with HTT-Interactors *
MID1, IKBKB, IKBKG	Negative	PPP2CA	≥0.72	Yes	402 (20)
		TUBB	≥0.63	Yes	81 (41)
		HSP90AA1	≥0.63	Yes	51 (47)
		RPS3	≥0.63	No	295 (23)
PML, OPTN, CHUK	Positive	HSPA9	≥0.49	No	47 (48)

* Out of 10,914 proteins. Number of interactions with HTT interactors indicated in parentheses.

## Data Availability

Not applicable.

## Supplementary Materials

The following supporting information can be downloaded at: https://www.mdpi.com/article/10.3390/ijms23105853/s1.
