# Supplementary material for: Analysis of Huntington’s Disease Modifiers Using the Hyperbolic Mapping of the Protein Interaction Network
Source: Int J Mol Sci. 2022 May 23;23(10):5853. doi: 10.3390/ijms23105853 (PMC9144261; doi:10.3390/ijms23105853)
Supplement: Supplementary file 1 [file ijms-23-05853-s001.zip › Supplementary Legends.pdf]

# Supplementary Materials

**Title:** Analysis of Huntington's Disease Modifiers Using the Hyperbolic Mapping of the Protein Interaction Network
**Authors:** Aimilia-Christina Vagiona, Pablo Mier, Spyros Petrakis and Miguel A. Andrade-Navarro

**Figure S1: Identification of large gaps between angular coordinates in the hPIN**. Proteins were sorted increasingly by their inferred angular coordinates θ and the difference between $\theta_i$ and $\theta_{i+1}$ was estimated. The peaks in the plot correspond to gap sizes in the angular dimension and uncover the presence of similarity-based clusters. The gap size $g$ = 0.011344 was chosen because it gives clusters with a minimum of 10 members.

**Figure S2: Identification of large gaps between angular coordinates in the hyperbolic disc of HTT interactors**. Proteins were sorted increasingly by their inferred angular coordinate θ and the difference between $\theta_i$ and $\theta_{i+1}$ was estimated. The gap size $g$ = 0.059198 was chosen. The peaks in the plot correspond to gap sizes in the angular dimension and uncover the presence of angular similarity-based clusters.

**Table S1: Hyperbolic coordinates of each protein of the hPIN**. Columns indicate protein identifiers (UniprotKB, Symbol), coordinates (r, theta) and cluster number.

**Table S2: HTT interactors in the hPIN (HttPIN)**. Columns indicate protein identifiers (UniprotKB, Symbol), coordinates (r, theta) and cluster number. Clusters were recomputed using only the HttPIN (see text for details).

**Table S3. Paralog pairs of the HttPIN**. Columns indicate identifiers and cluster of the first protein (Interactor UniprotKB, Interactor Symbol, Cluster interactor) and of the second protein (Paralog UniprotKB, Paralog Symbol, Cluster paralog). The order of the proteins in the pairs is arbitrary.

**Table S4: Paralog pairs of the HttPIN in different clusters and their effect on HD**. Columns indicate identifiers, cluster, effect and references of effect on HD of the first protein (Interactor UniprotKB, Interactor Symbol, Cluster interactor, Role interactor, References role interactor) and of the second protein (Paralog UniprotKB, Paralog Symbol, Cluster paralog, Role paralog, References role paralog). The order of the proteins in the pairs is arbitrary.
