# Supplementary figures and images for: Analysis of Huntington’s Disease Modifiers Using the Hyperbolic Mapping of the Protein Interaction Network
Source: Int J Mol Sci. 2022 May 23;23(10):5853. doi: 10.3390/ijms23105853 (PMC9144261; doi:10.3390/ijms23105853)

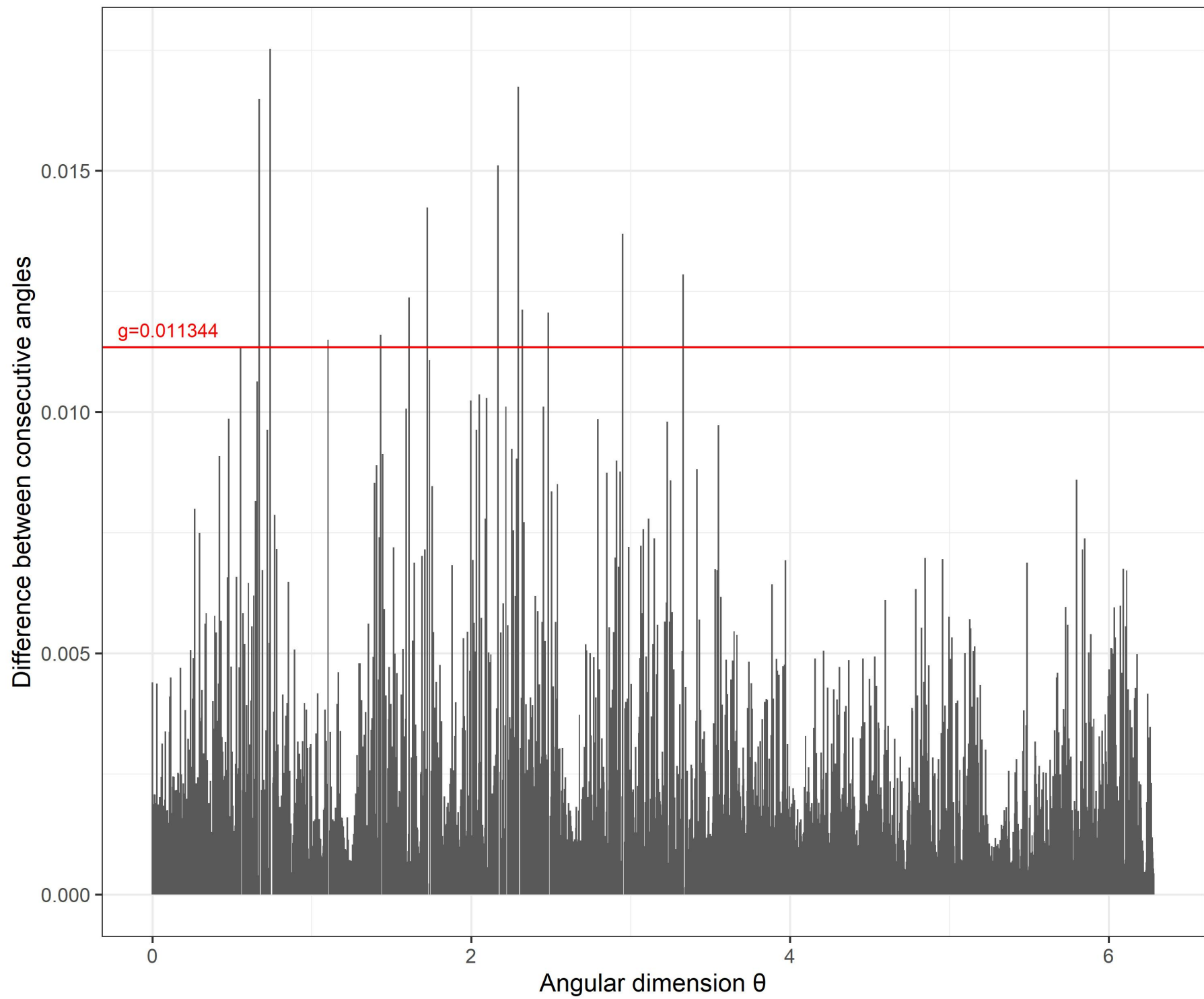

Supplement: Supplementary file 1 [file ijms-23-05853-s001.zip › Supplementary Figure S1.pdf]

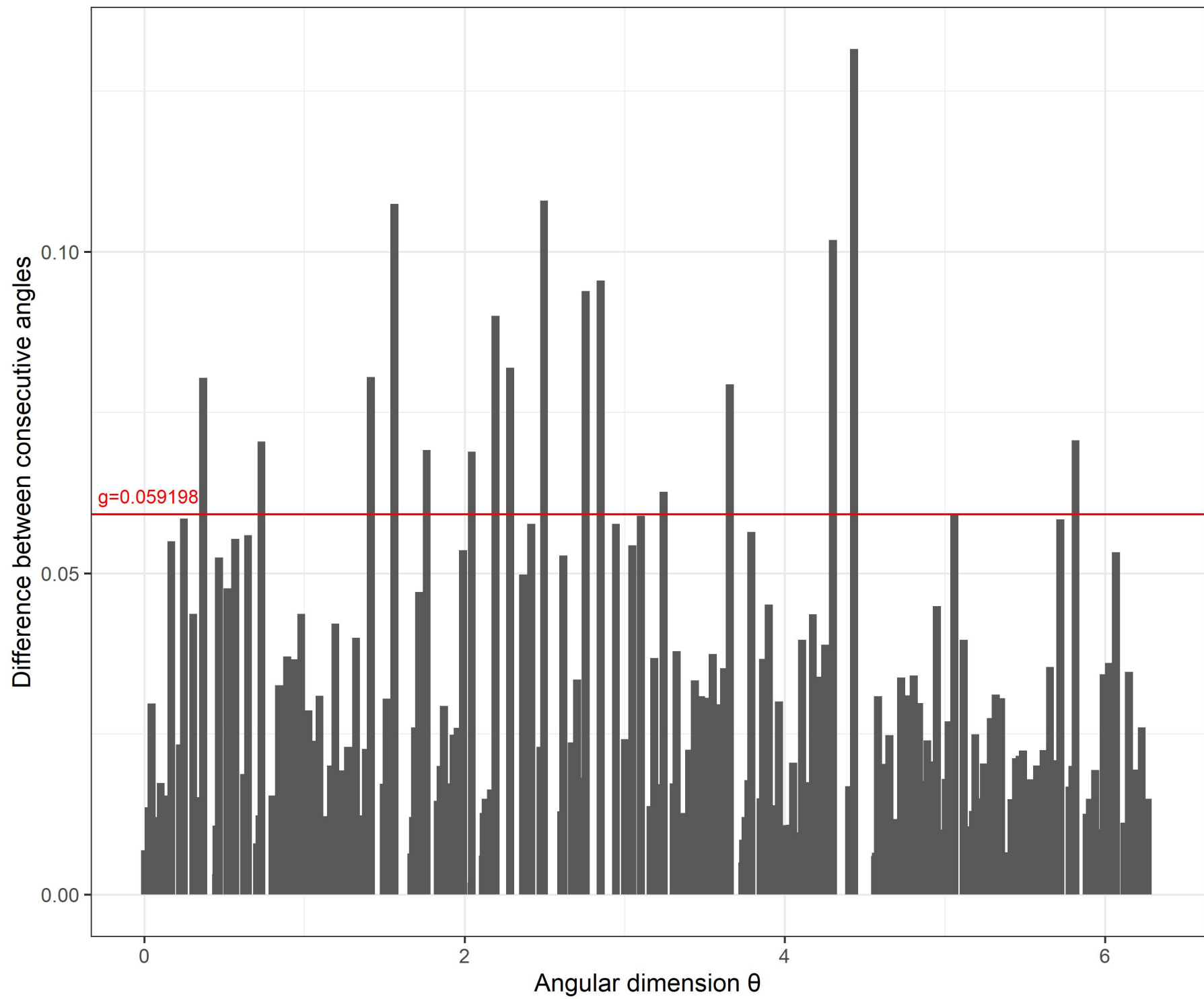

Supplement: Supplementary file 1 [file ijms-23-05853-s001.zip › Supplementary Figure S2.pdf]
